# Wealth, Health, and Disability Gaps at Midlife: Implications for Disparate Retirement Outcome

**DOI:** 10.1093/geroni/igaa057.1960

**Published:** 2020-12-16

**Authors:** Stephen Crystal

**Affiliations:** Rutgers University, New Brunswick, New Jersey, United States

## Abstract

Late-life economic outcomes in coming years will be strongly shaped by the impact of economic and health experience for cohorts now at midlife. These cohorts have experienced lagging and increasingly disparate wealth accumulation, along with challenges to health and earning potential that augur highly disparate retirement futures. For example, analyses of Survey of Consumer Finances data indicate that in 2016, members of Generation X had mean assets that were only 39% those of the boomers in that year. Increasing risk of “deaths of despair” among individuals in midlife have been accompanied by increases in disability that threaten the ability of those members of these cohorts who are not in the educationally advantaged minority to achieve secure retirement futures, particularly in the context of increasing employment precarity. This presentation will review recent findings on midlife wealth and health inequality, implications for retirement futures, and policy choices facing a new Presidential administration.

